# Inequality in the Incidence of Cervical Cancer: Costa Rica 1980–2010

**DOI:** 10.3389/fonc.2018.00664

**Published:** 2019-01-10

**Authors:** Carolina Santamaría-Ulloa, Cindy Valverde-Manzanares

**Affiliations:** ^1^Health Research Institute, University of Costa Rica, San José, Costa Rica; ^2^Health Surveillance Department, Ministry of Health Costa Rica, San José, Costa Rica

**Keywords:** developing countries, cervical cancer, social determinants, inequality, Costa Rica

## Abstract

**Introduction:** Cervical cancer is the third most incident and the fourth most lethal cancer among Costa Rican women. The purpose of this study was to quantify incidence inequality along three decades and to explore its determinants.

**Materials and Methods:** This is a population-based study. Main data sources were the National Tumor Registry (1980–2010), CRELES (Costa Rican Longevity and Healthy Aging Study) longitudinal survey (2013), and published indices of economic condition (2007) and access to healthcare (2000). Cartography was made with QGIS software. Inequality was quantified using the Theil-T index. With the purpose of detecting differences by tumor's behavior, inequality was estimated for “*in situ*” and invasive incidence. *In Situ*/Invasive Ratios were estimated as an additional marker of inequality. Poisson and spatial regression analyses were conducted with Stata and ArcMap software, respectively, to assess the association between incidence and social determinants such as economic condition, access to healthcare and sub-utilization of Papanicolaou screening.

**Results:** As measured by Theil-T index, incidence inequality has reached high (83 to 87%) levels during the last three decades. For invasive cervical cancer, inequality has been rising especially in women aged 50–59; increasing from 58% in the 1980's to 66% in 2000's. Poisson regression models showed that sub-utilization of Papanicolaou smear was associated with a significant decrease in the probability of early diagnosis. Costa Rican guidelines establish a Pap smear every 2 years; having a Pap smear every 3 years or longer was associated with a 36% decrease in the probability of early “*in situ*” diagnosis (IRR = 0.64, *p* = 0.003) in the last decade. Spatial regression models allowed for the detection of specific areas where incidence of invasive cervical cancer was higher than expected.

**Conclusion:** Results from this study provide evidence of inequality in the incidence of cervical cancer, which has been high over three decades, and may be explained by sub-utilization of Papanicolaou smear screening in certain regions. The reasons why women do not adequately use screening must be addressed in future research. Interventions should be developed to stimulate the utilization of screening especially among women aged 50 to 59 where inequality has been rising.

## Introduction

In 2005, the World Health Organization (WHO) created the Commission on Social Determinants of Health with the purpose of helping nations to face the social causes of health and reduce inequity (1). An explanatory model of the way by which health status is produced or affected within a population points to four categories of determinants: biological, environmental, health-service related and socio-economic as well as cultural (2).

The nature and the magnitude of inequality on health outcomes need to be investigated. On one hand, the nature refers to the origin of each situation; it allows to better understand the ways in which differences developed. On the other hand, assessing the magnitude may be associated with the impact this situation has on a population. Quantifications of inequality are used in the current study and further discussed under the framework of the social determinants of health to examine the incidence of cervical cancer as a health outcome.

Cervical cancer is the third most incident malignant tumor among Costa Rican females, with a rate of 30 cases per 100,000 women in 2015. This incidence is only surpassed by skin and breast cancer with rates of 61 and 60 cases per 100,000 women, respectively (3). It is the fourth most important cause of malignant cancer mortality among the female population with a rate of 6 deaths per 100,000 women in 2016. Cervical cancer mortality is only surpassed by breast, stomach, and colon cancer (4).

In Costa Rica, like in other countries, an association between cervical cancer incidence and geographic location has been previously described. Using data from the National Tumor Registry for the period 1980 to 1983, [Sierra and Barrantes (5)] found a higher incidence of cervical cancer in the coastal vs. non-coastal areas. Further, the Ministry Health (3) documented higher prevalences of cervical cancer in coastal regions. The geographical distribution of incidence shows that Costa Rican women are not affected in a homogeneous manner within the country.

The human papilloma virus (HPV) is the most important risk factor for the development of cervical cancer (6). The 2015 Costa Rican National Survey on Reproductive and Sexual Health indicates that in the country only 45% of women and 44% of men recognize the HPV as a sexually transmitted infection (7). Sexual behavior is an important risk factor for cervical cancer and socioeconomic status has been associated with sexual behaviors that favor the acquisition of HPV. [De Sanjosé and collaborators (8)], in two case-control studies carried out in Colombia and Spain, determined that human papilloma virus was more frequent in women of low vs. high socio-economic status. Moreover, another study confirmed the association between socioeconomic status and HPV for different cancer sites, including cervical cancer (9). However, higher prevalence of HPV in middle and high socio-economic status in Latin America and in developed countries, may evidence that socioeconomic differences in incidence result from access to screening inequality rather than HPV prevalence inequality. Sancho-Garnier et al. (10), make a case of how high income countries with established preventive programs have persistent inequalities in detection because of inequality in access to screening programs.

Starting in the 1960s and up to the 1980s, cervical cancer cases were primarily detected via population-based screening programs such as Papanicolaou smear, a conventional cytology that tests for the presence of precancerous or cancerous cells on the cervix. More recently, screening by human papillomavirus (HPV) testing has been established as more accurate and effective. Although HPV testing is expected to become the preferred screening test in the medium and long term (11), Pap smear is still the recommended screening procedure in healthcare protocols in Costa Rica. Timely access to screening services allows early detection of malignant tumors. In general, low-income women show higher rates of cancer detection at advanced stages (12). It is therefore frequently assumed that inequality in cervical cancer incidence in diverse populations may be the result of unequal access to screening services. If this were indeed the case, it would be expected that a public policy to improve access to screening would lead to a reduction of inequality (13).

In an exploratory analysis using geographic information systems and data from the National Tumor Registry between 1990 and 1997, [Santamaría (14)] reported a significantly higher incidence of cervical cancer in southern and Caribbean regions of the country, primarily in the provinces of Puntarenas and Limon, where the relative risk of invasive cervical cancer reached values that were 2.1 times higher than in the rest of the country. These regions have the lowest indices of human and social development in Costa Rica.

There is evidence that cervical cancer affects Costa Rican women in a heterogeneous manner; and it is also evident that this phenomenon has persisted for several decades in the country. Nevertheless, neither the magnitude of this existing inequality has been quantified, nor has the research on inequality been approached from the perspective of the social determinants of health. The aim of this study is to determine the magnitude of the disparities in cervical cancer incidence within Costa Rica and to identify factors associated with incidence, in order to inform policies to reduce disparities.

## Methods

This study was conducted after obtaining approval from the Scientific Ethics Committee at the University of Costa Rica (VI-3621-2012). This is a population-based study, geographic units rather than individuals are its units of analysis. Costa Rica has an area of 51,100 km^2^, administratively divided into 7 provinces. In 2018, a total of 82 counties and 484 districts were contained within those provinces. Cervical cancer incidence was analyzed both at the county and the district level for a 31-year time period: 1980 to 2010.

Analyses were conducted for total, *in situ*, and invasive cervical cancer incidence. Most of analyses were broken down into 3 periods: 1980–1989, 1990–1999, and 2000–2010. Regression models were estimated for the last period: 2000–2010.

### Data Description

Five sources of information were used: (1) Cancer cases from National Tumor Registry, (2) Population exposed from Official Population Estimations, (3) Population economic condition from the Social Development Index, (4) Population access to healthcare from a geographical access to healthcare index, and (5) Papanicolaou sub-utilization from the survey CRELES: Costa Rican Longevity and Healthy Aging Study.

Cancer cases come from the National Tumor Registry (NTR) database from 1980 to 2010. Access to the NTR was provided by the Costa Rican Ministry of Health. The standard NTR record contains age, calendar year, and place of residence at diagnosis. A count of cases for each geographical unit was made for each of the time periods of interest. This nationwide population-based registry has been maintained by the Ministry of Health since 1977. Since 1980 all hospitals and private pathologists have agreed to report any hospitalizations or outpatient biopsies associated with a cancer diagnosis (15). This registry has high indices of data quality (16) and since the 1980s this NTR's coverage has been estimated to be around 98% (17).

Population exposed from 1980 to 2010 within each geographical unit was estimated based on the official updated estimation figures for female population. These estimations are jointly elaborated by the National Institute for Population Statistics and Census and the Central American Population Center of the University of Costa Rica (18).

Using data from the NTR on newly diagnosed cases of cervical cancer as well as the official population estimations, we estimated cervical cancer incidence rates. Data from the NTR constitutes the numerator of the cervical cancer incidence rate for each geographic unit. The denominator of the rate is the female population at mid-period, multiplied by the number of years included in the numerator, in order to estimate annualized rates. Because age distribution in this population was not significantly different from Segi's world standard population (chi = 128, *p* = 0.292), crude incidence rates per 100,000 women were estimated for this study. QGIS software (19) was used to represent incidence rates in maps.

The following three data sources were used in regression models to explain incidence for the 2000–2010 period. Because of availability of data, they belong to different years, which are the closest possible to the 2000–2010 period.

Data on economic condition in 2007 is the official estimation of the Social Development Index' economic component, which is estimated by the Costa Rican Ministry of Planning. The economic dimension of the index is a compound measure of residential electricity consumption and residential access to Internet. It captures population capability of acquiring goods and services, and population's technology access (20).

Access to healthcare in 2000 is measured as a comprehensive index of geographic accessibility to healthcare facilities in Costa Rica. All healthcare facilities are included in this index: primary healthcare facilities, clinics and hospitals. It was created using Geographic Information System (GIS) technologies and aggregating characteristics of both population and healthcare facilities (21).

Sub-utilization of Papanicolaou screening in 2013 is estimated from the survey CRELES: Costa Rican Longevity and Healthy Aging Study. Data from this longitudinal survey is publicly available (22).

### Statistical Analyses

The Theil T index (23) was used to quantify inequality at a district level. Incidence rates were the basis for the quantitative estimation of inequality in the distribution of this pathology. This index has been widely used to measure inequality in different health and social outcomes. It has for example been used to measure income inequality in Latin America (24) or inequality in access to improved water in different world regions (25).

This indicator was selected because it can estimate inequality even when geographical units have a null incidence rate. Having a number of geographical units with no cases is an expected scenario given the small size of the unit of analysis (district) and the fact that the event is considered to be infrequent. Theil-T is a population weighted index that is sensitive to health differences further from the average rate (26).

The Theil-T index is defined as follows:

(1) $$\begin{array}{l} T=\sum_{u=1}^{N}y_{u}\log\frac{y_{u}}{\frac{1}{N}} \end{array}$$

Where:

For each *u* = 1,2,…, geographical units (districts)

*y*_*u*_ = number of cases of cervical cancer diagnosed in district u

*N* = female population size.

Carcinoma *in situ*/Invasive Cervical Cancer Ratios (CIS/ICC) were estimated as an additional marker of inequality. A CIS/ICC = 1 means that for each carcinoma *in situ*, there is another invasive cervical cancer detected. Ideally the incidence of carcinoma *in situ* should exceed that of invasive cancer (CIS/ICC > 1), indicating a majority of cases being detected at an early stage (27). CIS/ICC were represented in maps using QGIS software (19).

Multivariate regression analyses were also carried out at the district level in the 2000–2010 period. Poisson and geographically weighted spatial regression models were estimated with Stata (28) and ArcMap (29) software respectively, to assess the association between incidence and social determinants such as economic condition, access to healthcare and sub-utilization of Papanicolaou smear. Cervical cancer counts and incidence rates were the dependent variable of Poisson and spatial regression models, respectively. The social determinants of this health outcome (economic condition, access to healthcare, and sub-utilization of Papanicolaou smear) were controlled for as independent variables in both types of models.

Because cervical cancer is an infrequent event, cases are assumed to be generated from a Poisson distribution. Poisson is adequate to model cases of infrequent illnesses with a small number of cases (30). When the dependent variable is a counting (number of new cervical cancer cases on each district), that takes the form of entire non-negative values, a Poisson specification is an improvement over Ordinary Least Squares (31). The Poisson distribution provides the probability of the number of events; and the parameters correspond to the expected number of occurrences as a function of the independent variables (32). This model was estimated using Stata software (28).

Using the count of cases as the dependent variable in Poisson regression models, suggests the need to control for female population exposed to cervical cancer in each district, because each count of cases refers to areas of different underlying populations. The observed number of cases *b*_*i*_ was the dependent variable, and the expected number of cases $b_{i}^{E}$ was the offset variable introduced in the right hand side of the model. The Poisson regression model is defined as follows:

(2) $$\begin{array}{l} b_{i}=P(b_{i},x) \end{array}$$

(3) $$\begin{array}{l} b_{i}^{E}=\sum (M_{i}x^{*}W_{i}^{S}x) \end{array}$$

Where:

*b*_*i*_ is the observed number of cases at location *i*;

*P* indicates a Poisson function;

*x* is the age group;

$b_{i}^{E}$ is the expected number of cases at location *i*;

*M*_*i*_ *x* is the observed population size in location *i* at age *x*; and

$W_{i}^{S}$ *x* is the incidence rate in the standard population at age x.

A geographically weighted regression was also carried out. This spatial regression tool is based on mathematical models that take into account spatial auto-correlation and it has been previously used in cancer research conducted in Costa Rica (33). Counties close to each other have a greater probability of sharing characteristics among themselves due to their geographic proximity than those that are located more distantly from one another, which makes this methodology relevant for the current study. This model was estimated using the GWR (Geographically Weighted Regression) tool in ArcMap software (29) and maps were made using QGIS software (19).

Parameters in a global regression model are very likely not constant across space, and geographically weighted regressions allow determining how each parameter varies across a geographical area. This statistical tool helps understand spatial heterogeneity in data (33), which justifies its use in this study.

Social determinants were used as independent variables in both the Poisson and the spatial regression model. The Costa Rican Ministry of Health (34), following Lalonde (2), classifies social determinants into four categories: (1) biological determinants; (2) environmental determinants; (3) socioeconomic as well as cultural determinants; and (4) determinants related to the healthcare services. Controlling for all four categories of determinants in regression models would be optimal. Nevertheless, at the population level there is no information regarding the first category of biological factors such as the population prevalence of human papilloma virus (HPV). There is also no data available on the second category of environmental factors that may be associated with the incidence of cervical cancer such as tobacco smoking (35).

Although for the purpose of this study it is not possible to explore the association between cervical cancer and biological and environmental factors, the third and fourth categories of determinants—socioeconomic and healthcare—have been included in the analyses.

Regarding the third category of socioeconomic determinants, it is desirable to consider several dimensions of socioeconomic status (36). The economics dimension of the social development index (SDI) was used in this study as a measure of economic condition at the district level (20). This approach of quantifying socioeconomic determinants is similar to the one used in a previous research (37).

Regarding the fourth category of determinants related to the healthcare services, density index of access to healthcare services and sub-utilization of Papanicolaou smear were included.

Although access to healthcare is a concept with at least two dimensions: geographic and social (38), geographic access is what this index measures. Geographical access to healthcare facilities in 2,000 is measured using a comprehensive index of accessibility that results from the aggregation of all facilities weighted by their size, proximity, and characteristics of both the population and the facility. The density index of access to healthcare services uses physician hours per capita yearly as the metric. The greater the value a district has for this index, the better access to health services has its population. Greater details on the construction of this index can be found in Rosero-Bixby (21).

Sub-utilization of Papanicolaou screening in 2013 was obtained from CRELES: Costa Rica Study of Longevity and Healthy Aging. Rates are based on a question about when was the last time women had a Papanicolaou screening. According to national attention guidelines, Papanicolaou screening should be conducted at least every 2 years (39). A measure of the proportion of female population who had their last screening 3 years ago or longer is used as an indicator of sub-utilization of Pap smear in this study. Because of sampling issues, it was not possible to obtain district level estimations; therefore the indicator was estimated for counties, which are larger geographic units.

## Results

A total of 22,279 incident cases of cervical cancer occurred in Costa Rica during the 1980–2010 time period. Because this study is based on geographical units, 5.4% of cases were not included on the grounds of not containing any information about patient's place of living at diagnosis.

A total of 21,075 cases were included in the analyses. In absence of information, district or county imputation was conducted in 7.0% of cases (5.3% of cases with no district information and 1.7% of cases with no county information). Imputation was made under of the assumption that missing information followed the distribution of non-missing cases within its corresponding county or province.

Cases were distributed along the period of study as follows: 26% during 1980–1989, 31% in 1990–1999, and 42% in 2000–2010. The number of cases had a 79% increase from the 1980s to the 2000s, which is mainly attributed to an increase in the detection of carcinoma *in situ*. Details on numerators, denominators, incidence rates, and ratios by period are included in Table 1.

**Table 1:** Descriptive data on cervical cancer incidence, by time period. Costa Rica: 1980–2010.

**Indicator**	**1980–1989**	**1990–1999**	**2000–2010**	**Total**
**CERVICAL CANCER CASES**				
Excluded from analyses				
No geographical location available	595	47	562	1.204
Included in analyses				
*In situ*	2,998	3,421	6,202	12,621
Invasive	2,239	2,559	3,656	8,454
Total cases included in analyses	5,237	5,980	9,858	21,075
Total cases reported in NTR	5,832	6,027	10,420	22,279
**POPULATION EXPOSED**				
Female population at mid-period	1,281,313	1,671,216	2,065,853	1,671,216
**CERVICAL CANCER ANNUALIZED INCIDENCE RATE PER 100,000 WOMEN**				
*In situ*	23.40	20.47	27.29	24.36
Invasive	17.47	15.31	16.09	16.32
Total	40.87	35.78	43.38	40.68
*In situ*/Invasive Incidence Rate Ratio	1.34	1.34	1.70	1.46

Inequality was analyzed by first describing geographical differences in the incidence of cervical cancer and then measuring the association between such health outcome and its social determinants as an approach to hypothesize on explanations to disparities. The estimation of the degree of inequality was carried out for each of the three decades and it was also analyzed by tumor behavior (*in situ* or invasive) with the purpose of determining the existence of differences. Analyzing results by tumor behavior is meaningful for understanding health disparities because behavior is itself an indication of how timely the cancer was diagnosed.

**Figure 1:**
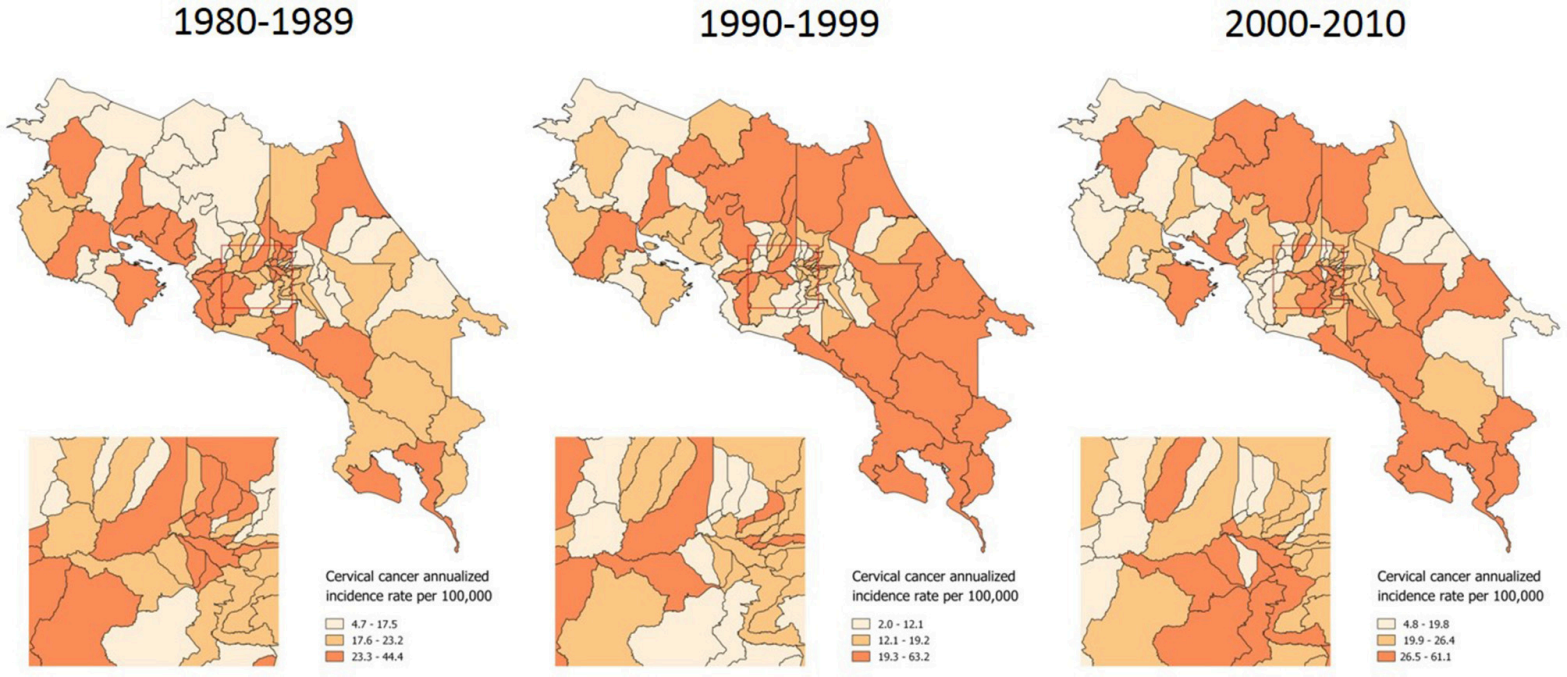
Incidence of *in situ* cervical cancer, by time period. Costa Rica: 1980–2010 (Annualized rates per 100,000 women).

In the first phase of the analysis, a description of inequality was made by using cartographic representations and by estimating the Theil-T index for incidence of cervical cancer in Costa Rica. Across three decades, the incidence of carcinoma *in situ* has been heterogeneously distributed along the territory, although in terms of territory extension the 1990s had the greatest area of high *in situ* cases incidence rates (Figure 1).

**Figure 2:**
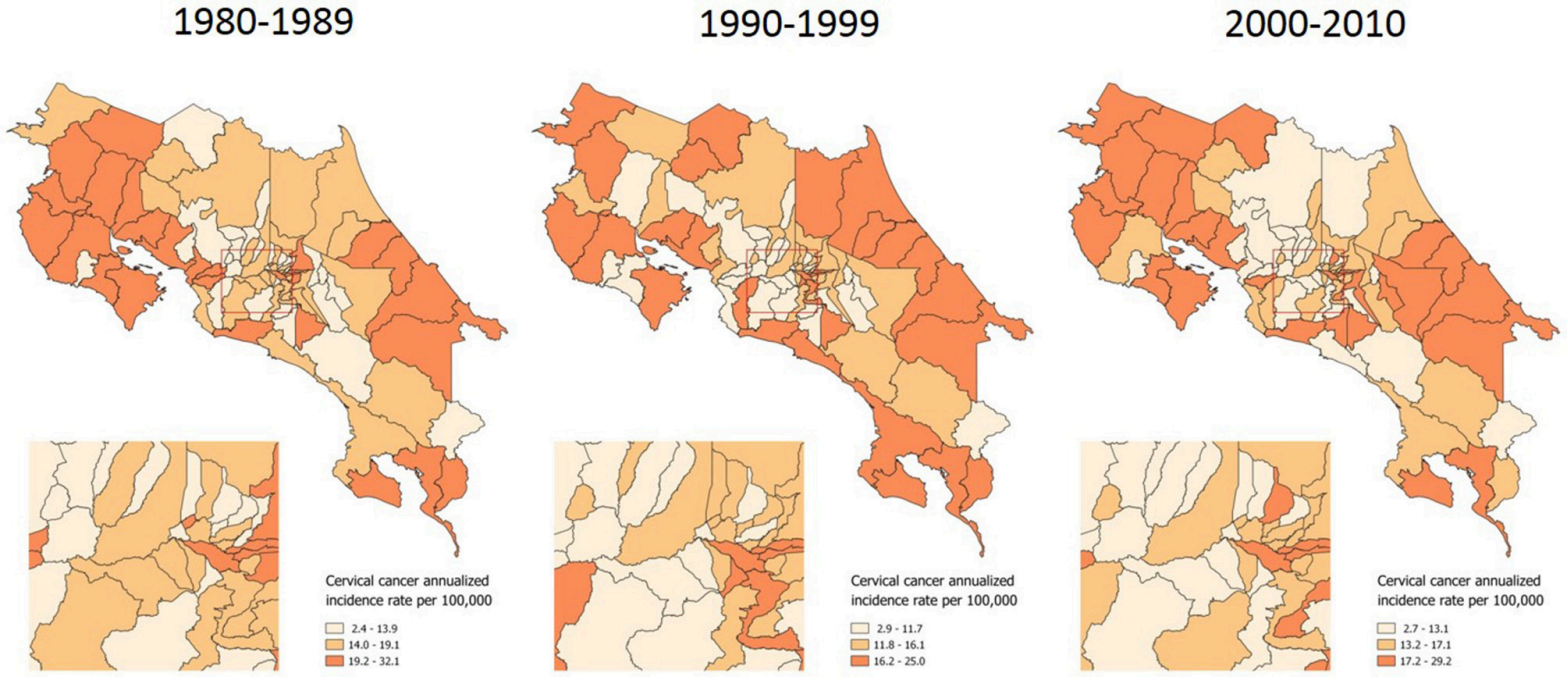
Incidence of invasive cervical cancer, by time period. Costa Rica: 1980–2010 (Annualized rates per 100,000 women).

A distribution pattern is more evident when examining the incidence of invasive cervical cancer, which has more clearly concentrated in the country's coastal and border areas along the last 31 years (Figure 2).

**Figure 3:**
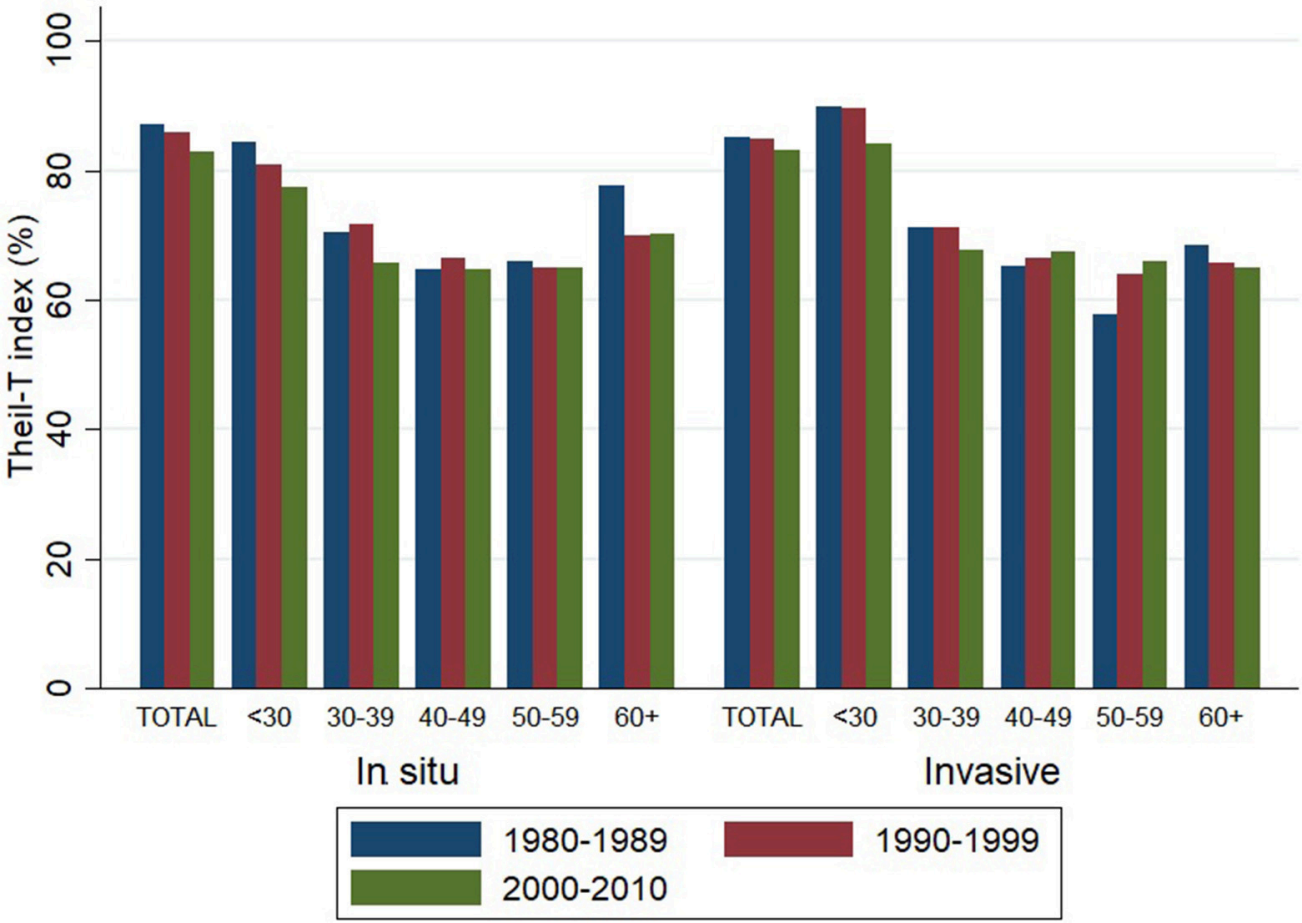
Inequality in the incidence of cervical cancer, by tumor behavior and time period. Costa Rica: 1980–2010 (Theil-T Index, %).

Theil-T index values are estimated on a scale that ranges from zero to 100%, where zero is perfect equality and 100% is perfect inequality. As measured by the Theil-T index, inequality has moved from 87 to 83% and from 85 to 83% for *in situ* and invasive cervical cancer, respectively, along three decades (Figure 3). All of these values over 80% are evidence of high inequality levels. But in spite of this rather high level of inequality, two important phenomena have taken place. On one hand, inequality of *in situ* cervical cancer has decreased in the population younger than 40 (left hand side of Figure 3).

On the other hand, inequality in invasive cervical cancer has increased in the older population aged 40 to 59, but especially in the 50–59 age group (right hand side of Figure 3), where the inequality increased 11% from the 1980s to the 1990s and reached a total 14% increase during the 31 year period from 1980 to 2010 (Table 2). The increase in inequality observed for invasive cancer in women aged 50–59 was greater than any decrease in inequality observed in other age groups (Table 2).

**Table 2:** Relative (%) change in inequality as measured by Theil-T index in the incidence of cervical cancer, by tumor behavior and period of change. Costa Rica: 1980–2010.

**Age**	***In situ***			**Invasive**		
	**1980s−1990s**	**1990s−2000s**	**1980s−2000s**	**1980s−1990s**	**1990s−2000s**	**1980s−2000s**
<30	−4.0	−4.2	−8.0	−0.5	−6.2	−6.6
30–39	1.7	−8.1	−6.6	0.2	−5.1	−4.9
40–49	2.6	−2.7	−0.2	1.6	1.8	3.4
50–59	−1.6	0.1	−1.5	10.7	2.9	13.9
60+	−10.0	0.5	−9.6	−4.0	−1.1	−5.0
Total	−1.2	−3.7	−4.8	−0.2	−2.2	−2.4

Carcinoma *in situ*/Invasive Cervical Cancer Ratios (CIS/ICC) are also presented as indicators of inequality. They have the advantage of allowing a cartographic representation of geographical areas where inequalities occur. In Costa Rica, the CIS/ICC ratio averaged 1.46 from 1980 to 2010 (Table 1). Results from this indicator were presented in maps with ratios divided into three categories: <1 in red color (mostly late detection), 1–1.49 in white color (around national average ratio), and > 1.50 in blue color (mostly early detection).

**Figure 4:**
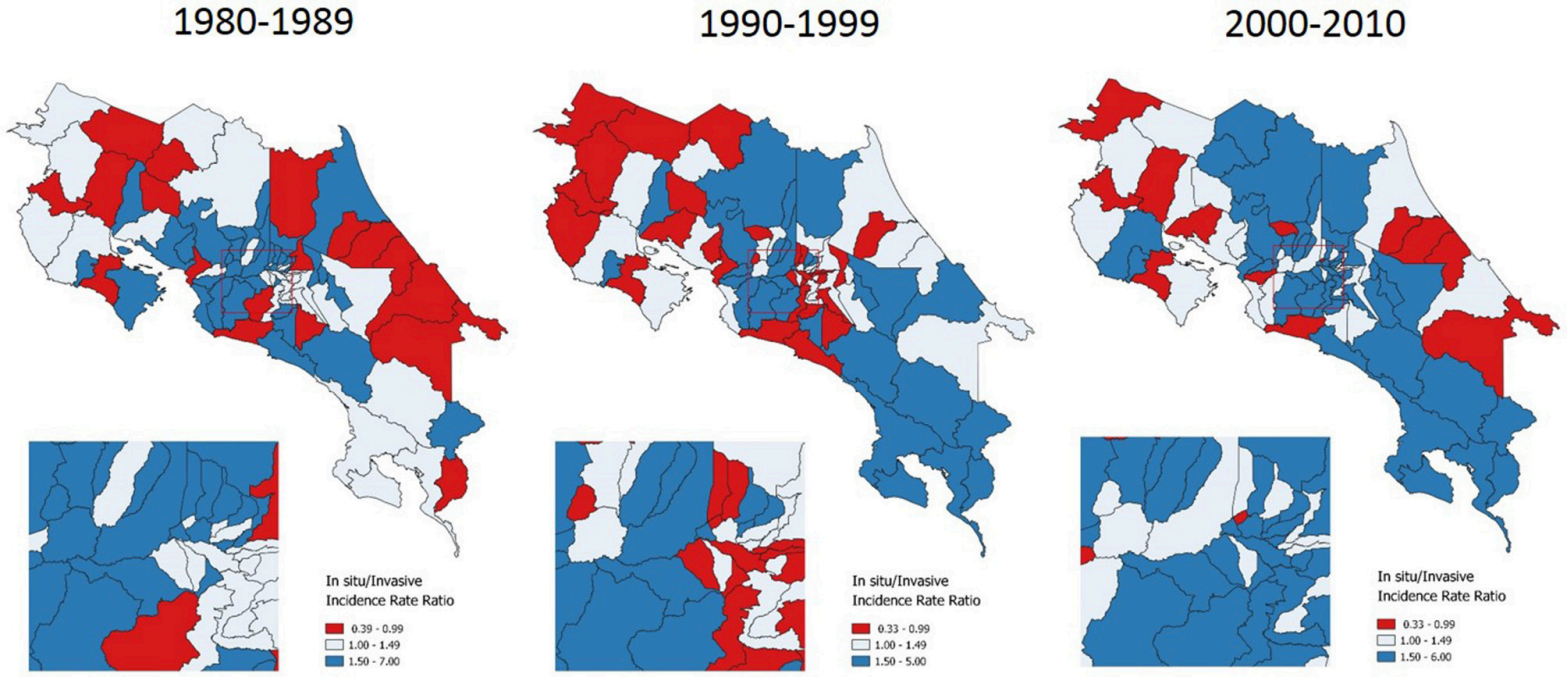
*In situ*/Invasive Cervical Cancer Incidence Ratios, by time period. Costa Rica: 1980–2010.

CIS/ICC shows a concentration of red geographical units in border areas, these are areas of late detection for cervical cancer, meaning that a majority of new cases are diagnosed in late stages rather than *in situ*. This inequality concentrates to the North where border is shared with Nicaragua, to the South where Costa Rica shares a limit with Panama, and in some coast areas (Figure 4).

In the second phase of the analysis, Poisson and spatial regression models were estimated to measure the association between incidence and its social determinants. This was done as an exercise to generate hypotheses about the factors that may explain the inequality found in the first phase of analysis.

Poisson regressions were modeled at the geographical district level. Incidence for the 2000–2010 period was modeled as a function of economic condition, geographical access to healthcare facilities and sub-utilization of papanicolaou screening, which were in turn proxy measures of the social determinants of incidence.

These Poisson regression analyses were conducted for total incidence and they were also stratified by tumor behavior; that is, for *in situ* and for invasive diagnosis. Incidence Rate Ratios (IRR) were estimated. Sub-utilization of Papanicolaou was significantly associated with a 36% decrease in the probability of early “*in situ*” diagnosis (IRR = 0.637, *p* = 0.003) (Table 3).

**Table 3:** Incidence rate ratios from a Poisson regression model to explain the incidence of cervical cancer, by tumor behavior. Costa Rica: 2000–2010.

**Independent variables**	***In situ***		**Invasive**		**Total**	
	**IRR**	**p**	**IRR**	**p**	**IRR**	**p**
Economic condition	1.002	0.175	1.008	<0.001	1.004	<0.001
Geographic access to healthcare	1.001	<0.001	1.001	<0.001	1.001	<0.001
Pap sub-utilization	0.637	0.003	1.020	0.921	0.755	0.022

**Figure 5:**
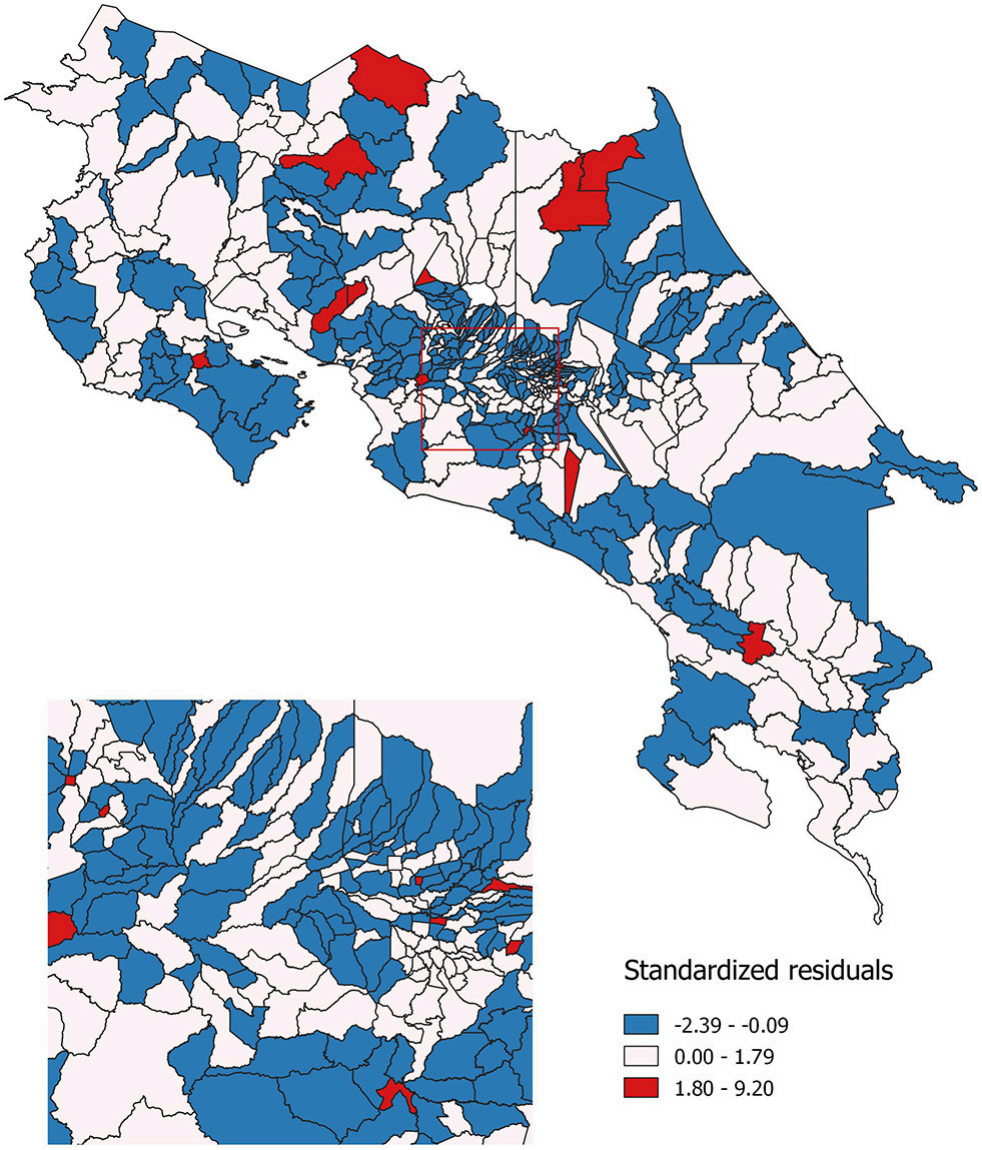
Observed as compared to expected probability of invasive cervical cancer incidence. Costa Rica: 2000–2010.

Finally, geographic regression analyses were conducted for invasive cervical cancer incidence. Same as with Poisson models, this regression was estimated for the 2000–2010 period. The same set of independent variables that were used in the previous models, was used for this spatial regression model: economic condition, geographical access to healthcare services and sub-utilization of Pap smear. Standardized residuals resulting from this modeling are shown in Figure 5. These residuals represent the difference between the observed incidence of invasive cervical cancer and the incidence that was predicted by the spatial regression equation. Areas where the incidence was lower than expected are represented in blue; areas where the incidence is approximately the same as expected are represented in white and those where the incidence is greater than expected are represented in red.

## Discussion

In Costa Rica the Cervical Cancer Prevention Program was created in 1960. Beginning the 1970s Papanicolaou screening was increasingly taking part of sexual and reproductive programs targeted to women 15 to 49 years of age. As a result, Pap smear coverage had an important upsurge. In the early 1980s, the national coverage of at least one Pap smear during lifetime was 51% for women aged 15–49, but it reached 70% in 1986 and 74% in 1993 (40–42). The geographical pattern observed in the incidence of *in situ* cervical cancer from the 1980s to the 1990s decade, when the greatest territory extension of *in situ* high rates occurred, illustrates the expansion of the screening program that has just been described.

Nonetheless, as Theil-T index results showed, the most relevant decrease in inequality occurred from the 1990s to the 2000s rather than from the 1980s to the 1990s. In 1995, a health sector reform was initiated in lower socioeconomic regions of the country, and it was progressively expanded to the entire country. This reform implied a better allocation of resources given the fact that instead of having two government institutions providing services, the Social Security System was assigned to offer healthcare services and the Ministry of Health was assigned a directing role. Rosero-Bixby (43) showed that this reform had an impact on reducing inequality in access to primary healthcare services. Our study findings support Rosero-Bixby's conclusion of a decrease of inequality in cervical cancer incidence from the 1990s to the 2000s that is probably a result of the combination of a well-established Cervical Cancer Prevention Program in the context of a health sector reform. This inequality decrease however, did not occur for all age groups and geographic areas of the country.

Female population younger than 40 years experienced the benefits of a national prevention program that was probably more successful in screening women at reproductive age, than it was in following-up and treating, especially after childbearing. Inequality in cervical cancer showed a modest decrease in 31 years, a decrease that could have been more important had older age groups received equal benefits that were received by younger women. In this context, although it is known that after the age of 40 the risk of cervical cancer decreases, that is the age that signals inequality rises in Costa Rica. Women aged 50 to 59 is the worst off group. The increase in inequality in this group is greater than any gain in terms of equality occurred in the rest of age groups along three decades. These disadvantaged women belong to cohorts that, in terms of age, either were part or soon became part of the prevention program target population. In the 1960s the prevention program started. Women aged 50 to 59 in the 1980s were around 30 back then. Those aged 50 to 59 in the 1990s were about 20 when the prevention program started. And those aged 50 to 59 in the 2000s were around 10 years of age. These cohorts probably experienced the advantages of the prevention program in terms of screening, but also experienced the disadvantages of lack of follow-up. Not only these cohorts have not had a decrease in the *in situ* incidence inequality, but also have had the most important inequality increase in invasive cervical cancer, representing lost opportunities in cancer prevention.

In terms of geographic inequality, coast areas have long been described as the highest incidence regions the country. Between 1980 and 1983 Guanacaste and Puntarenas, both of them coast provinces, were shown to have the highest incidence; and Limón, another coast province, had the highest mortality. Unequal access to screening in Limón, as well as sexual behavior patterns in the coast were hypothesized as possible causes of geographic inequality (42). Between 1986 and 1987, (44) conducted a case-control study and concluded that the higher incidence of cervical cancer in coastal vs. metropolitan areas could not be attributed to differential access to Papanicolaou screening but to differences in reproductive behavior among populations. They observed these differences in age at first intercourse, number of sexual partners, number of children, and history of sexually transmitted infections, among others. (45) showed how Limón is a province of high inequality in terms of cervical cancer, with the highest share of invasive as compared to *in situ* incidence.

Our study findings regarding the geographical pattern of invasive cervical cancer show again how coast areas continue having the highest rates, as well as the lowest *In situ*/Invasive Cervical Cancer Ratios along three decades. Given the lack of population based data, testing an association between sexual behavior differences (42, 44) and incidence rates was not feasible in this study. However, an association between screening sub-utilization and incidence was found. The fact that after controlling for the effect of both economic condition and geographical access to healthcare services, screening sub-utilization is significantly associated with a lower probability of early detection is an important finding.

Previous studies have also found an association between cervical cancer incidence and low utilization rates of screening (46, 47). It has been described that cultural and social values are factors that influence access to cervical cancer screening (48). Future research should address the reasons why in a universal healthcare system such as the Costa Rican one, women still do not adequately access Papanicolaou screening. Geographical access according to our study can be ruled out, but cultural aspects may be mediating decisions to access screening services. This study results highlight finer tuned places where more research should be conducted to explain an incidence of invasive cervical cancer that exceeds what could be predicted.

## Conclusion

An unequal distribution of cervical cancer incidence has been described around the world. Disparities resulting from unnecessary, avoidable and unjust inequality occur globally (49). Although cervical cancer mortality rates have decreased over time, inequality has persisted in different contexts all over the globe. Taken altogether this study results provide evidence of inequality and highlight age groups and geographical areas that merit special attention. Inequality in the incidence of cervical cancer must be avoided regardless of women's age or place of residence. Age groups where inequality has been increasing and areas with a significantly higher than expected incidence of invasive cervical cancer represent opportunities to target early detection initiatives.

Most of cervical cancer cases may be detected with screening. Timely access to preventive services facilitates the detection of this neoplasm in early stages. Nevertheless, in general, low-income women have higher detection rates in late stages (12). In the United States, similar to what happens in Costa Rica and other countries; incidence has significantly decreased since the introduction of Papanicolaou screening. Nonetheless, even with the existence of screening, disparities in the incidence of cervical cancer persist in the US (50) as well as in other populations such as Costa Rica where a universal healthcare system is in place.

Cancer control and prevention are key to decrease inequality (51, 52). Response to cervical cancer can be divided into primary and secondary care. Focused on prevention, vaccination constitutes an advisable primary care strategy. Over the years, the use of the HPV vaccine has demonstrated to be an effective way to prevent cervical cancer. Including the HPV vaccine in the vaccination scheme has been previously suggested as a mean to improve the effectiveness of the Cervical Cancer Prevention Program in Costa Rica (45). Although the HPV vaccine is not yet available for the entire population in Costa Rica, it has recently been approved to be included in the social security system's vaccination scheme starting in 2019 in 10-year-old girls.

Secondary care is based on two elements, early diagnosis, and screening. Improving detection and offering opportune treatment of diagnosed cases are necessary conditions to alleviate the cervical cancer burden. Differences in Pap screening procedures among regions within the country and long waiting times between sampling and availability of laboratory results have been previously described as critical points in the Cervical Cancer Prevention Program in Costa Rica (53). Since the 1990s, human papillomavirus HPV-DNA testing has been proposed for the detection of cervical cancer precursors, either as a complement or as an alternative method to Pap smear. Epidemiological studies in Costa Rica and other developing countries have evidenced the effectiveness of HPV-DNA testing (54–56). [Quirós (45)] has suggested the inclusion of HPV-DNA testing in the Cervical Cancer Prevention Program in Costa Rica, which may be of special interest in the context described in this study.

Once inequality exists, it can only decrease if actions are taken toward such purpose. Policy aimed at specifically diminishing inequality in cervical cancer incidence is warranted. Results from this study identify regions of the country where actions may be focused in order to reduce gaps in women's health. Populations in the coast and border regions of the country should be prioritized. Integrated and inter-institutional approaches to education and health promotion are recommended. Strategies to promote an adequate use of screening with priority among women aged 50 to 59 years should be established in Costa Rica.

## Author Contributions

CS-U: Study design, data analysis, manuscript writing; CV-M: Manuscript writing.

### Conflict of Interest Statement

The authors declare that the research was conducted in the absence of any commercial or financial relationships that could be construed as a potential conflict of interest.
